# Online Training on Workplace Education in End-of-Life Care for Home Care Educators: A Randomized Controlled Trial

**DOI:** 10.1093/geroni/igaf122.3629

**Published:** 2025-12-31

**Authors:** Ryousuke Yamada, Hanako Numata, Kosuke Kashiwabara, Maiko Noguchi-Watanabe, Yuka Sumikawa, Kayoko Kurita, Noriko Yamamoto-Mitani

**Affiliations:** University of Tokyo, Bunkyo, Tokyo, Japan; University of Tokyo, University of Tokyo, Tokyo, Japan; University of Tokyo Hospital, University of Tokyo Hospital, Tokyo, Japan; The University of Tokyo, Bunkyo, Tokyo, Japan; University of Tokyo, Bunkyo-City, Tokyo, Japan; University of Tokyo, Bunkyo, Tokyo, Japan; The University of Tokyo, Bunkyo, Tokyo, Japan

## Abstract

No well-established and effective evidence-based training programs exist for home-care nursing educators to provide workplace education in end-of-life (EOL) home care. We developed and evaluated an online training program for educators to improve their on-the-job training (OJT) in EOL home care. We conducted a randomized controlled trial involving 108 educators, assigning them to either an intervention or a waitlist control group. The program included a lecture, two case-study sessions, and a follow-up message. The primary outcome was the change in general EOL educators’ attitudes, as measured by the End-of-Life Nursing Education Questionnaire, while the secondary outcomes included changes in OJT-specific knowledge, attitude, and educational practices. We analyzed the data using analysis of covariance, adjusting for the baseline. The intervention group demonstrated significantly higher post-intervention scores in confidence in education (a primary outcome component; p < .001), and in the secondary outcomes of OJT attitude (p = .003), and practice (p < .001). No significant inter-group differences were found regarding educational motivation, perceived impact on learners (primary outcome components), or OJT-related knowledge. These findings suggest that a brief, structured online program can effectively improve educators’ confidence in EOL education and OJT-related attitudes, as well as their self-reported practice of OJT for EOL home care. This program offers a scalable solution for improving staff training quality in home-care nursing settings. This study was approved by the Research Ethics Committee of the Graduate School of Medicine, The University of Tokyo (Approval No. 2024359NI-[2]) and registered with the University Hospital Medical Information Network (UMIN000056165).

